# Development of drug loaded cardiovascular prosthesis for thrombosis prevention using 3D printing

**DOI:** 10.1016/j.msec.2021.112375

**Published:** 2021-10

**Authors:** Juan Domínguez-Robles, Tingjun Shen, Victoria A. Cornelius, Francesca Corduas, Elena Mancuso, Ryan F. Donnelly, Andriana Margariti, Dimitrios A. Lamprou, Eneko Larrañeta

**Affiliations:** a School of Pharmacy, Queen's University Belfast, Lisburn Road 97, Belfast BT9 7BL, UK; b Wellcome-Wolfson Institute for Experimental Medicine, Queen's University Belfast, Belfast BT9 7BL, UK; c Nanotechnology and Integrated Bio-Engineering Centre (NIBEC), Ulster University, Jordanstown Campus, Newtownabbey BT37 0QB, UK

**Keywords:** 3D printing, Biodegradable vascular grafts, Polycaprolactone, Dipyridamole, Antithrombotic effect

## Abstract

Cardiovascular disease (CVD) is a general term for conditions which are the leading cause of death in the world. Quick restoration of tissue perfusion is a key factor to combat these diseases and improve the quality and duration of patients' life. Revascularization techniques include angioplasty, placement of a stent, or surgical bypass grafting. For the latter technique, autologous vessels remain the best clinical option; however, many patients lack suitable autogenous due to previous operations and they are often unsuitable. Therefore, synthetic vascular grafts providing antithrombosis, neointimal hyperplasia inhibition and fast endothelialization are still needed. To address these limitations, 3D printed dipyridamole (DIP) loaded biodegradable vascular grafts were developed. Polycaprolactone (PCL) and DIP were successfully mixed without solvents and then vascular grafts were 3D printed. A mixture of high and low molecular weight PCL was used to better ensure the integration of DIP, which would offer the biological functions required above. Moreover, 3D printing technology provides the ability to fabricate structures of precise geometries from a 3D model, enabling to customize the vascular grafts' shape or size. The produced vascular grafts were fully characterized through multiple techniques and the last step was to evaluate their drug release, antiplatelet effect and cytocompatibility. The results suggested that DIP was properly mixed and integrated within the PCL matrix. Moreover, these materials can provide a sustained and linear drug release without any obvious burst release, or any faster initial release rates for 30 days. Compared to PCL alone, a clear reduced platelet deposition in all the DIP-loaded vascular grafts was evidenced. The hemolysis percentage of both materials PCL alone and PCL containing 20% DIP were lower than 4%. Moreover, PCL and 20% DIP loaded grafts were able to provide a supportive environment for cellular attachment, viability, and growth.

## Highlights

• 3D printing was used to prepare PCL-based small diameter vascular grafts loaded with dipyridamole.
• Drug loaded grafts provided resistance to platelet adhesion.
• The mechanical properties of the 3D printing materials are in line with materials used for cardiovascular applications.
• 3D printed grafts are hemocompatible and cytocompatible.

## Graphical abstract

**Figure:**
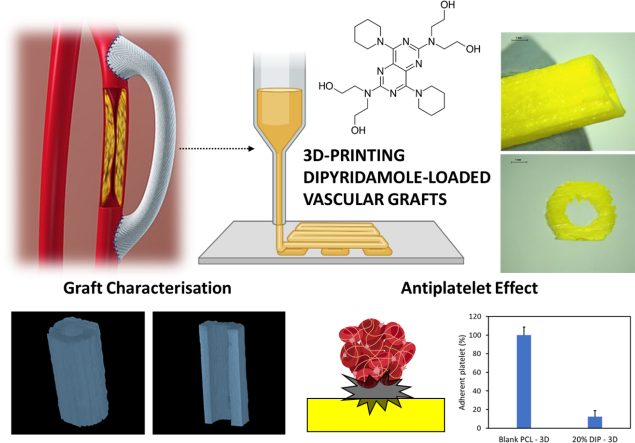

## 1 Introduction

Cardiovascular disease (CVD) is a general term used for conditions affecting the heart or blood vessels. The main types of CVDs include ischemic heart disease, heart failure, peripheral arterial disease and stroke [1]. CVDs remain the leading causes of death globally and represent a main contributor to reduced quality of life [2,3]. For instance, it has been estimated that 17.9 million peopled died from CVDs in 2016, representing 31% of all global deaths, and this burden is predicted to increase to 23.6 million death globally by 2030 [4,5]. Quick restoration of tissue perfusion is a key factor to prevent heart failures in people suffering from coronary heart disease and for supporting the repair of ischemic limbs. Revascularization techniques include angioplasty, placement of a stent, or surgical bypass grafting [6]. For the latter technique, autologous vessels remain the best clinical option, but the process of harvesting vessels is invasive, and they are often unsuitable [[7], [8], [9]]. Therefore, synthetic vascular grafts made from biocompatible materials are still needed [1,6].

These artificial vascular grafts offer advantages of flexibility in their shape, length, and diameter, which can be used in distinct practical settings [10,11]. Moreover, most of these are made from non-biodegradable materials, such as expanded polytetrafluoroethylene, polyethylene terephthalate and polyurethane [1,12]. Although these polymers are exceptional in their biocompatibility, chemical stability, low toxicity and long-term robustness, have a limited success when used for the replacement of small-diameter blood vessels (<6 mm internal diameter) [13,14] due to the unfavorable biological responses, including the formation of thrombosis, neointimal hyperplasia and delayed reendothelialization [[15], [16], [17]].

Attempts to overcome the limitations of these synthetic polymer grafts include embed them with antithrombotic drugs, vascular tissue engineering [7] or the development of new combinations of materials that promote the healing process and facilitate gradual degradation and complete replacement by natural blood vessels [12]. In this regard, polycaprolactone (PCL) is a biodegradable and hydrophobic synthetic polymer, which has been extensively used for cardiovascular applications due to its non-toxicity and biocompatibility, among other features [12,[18], [19], [20]]. Vascular grafts made from biodegradable polymers have the potential to regenerate blood vessels similar to those of patients and can be degraded, thus enabling a functional vascular replacement [[21], [22], [23]]. Moreover, this polymer presents a slow bioresorption kinetic (>1 year), which makes it a promising candidate for the development of tissue-engineered blood vessels, enabling seeded cells to produce extracellular matrix (ECM) and maintain a sustained drug release [19,20,24,25]. PCL has also been approved by the Food and Drug Administration (FDA) for its use as implantable biomaterial as well as in the development of drug delivery systems [26].

The combination of vascular grafts with antithrombotic drugs is a simple way to improve the biological functions of biodegradable polymers [1,19,21,27,28]. An ideal vascular graft should be able to prevent thrombosis and intimal hyperplasia, as well as promoting rapid endothelialization [21]. Although different antithrombotic drugs could be used for this purpose, dipyridamole (DIP), a clinically used drug for different cardiovascular conditions, has shown promising results reducing smooth muscle cell (SMC) proliferation [[29], [30], [31]] and promoting proliferation of vascular endothelial cells (EC) [31,32] in addition to its antithrombotic effect [33]. Different techniques including electrospinning [19,21,34] or thermally induced phase separation [35], among others, can be used to fabricate vascular grafts containing pharmaceutically active compounds. Alternatively, additive manufacturing (AM) can be used not only to prepare tailored vascular grafts personalized to the patient, but to include antithrombotic compounds to prevent the thrombosis associated with valvular prosthesis and vascular grafts [1].

AM, also known as 3D printing, is an increasingly growing manufacturing technology that provides the ability to fabricate structures of precise geometries from 3D model by the deposition of material in a layer-by-layer fashion. This technology has been previously investigated for the manufacture of multiple 3D devices medical devices including surgical meshes [[36], [37], [38]], subcutaneous Implants [39], wound dressings [40,41] or catheters [42,43] among others, showcasing the versatility of this technique for biomedical applications. In addition, cardiovascular prosthesis containing a small percentage of antimicrobial compounds to prevent infections have been performed using 3D printing technology [1]. The 3D printing approach can be used to produce biodegradable, biocompatible implantable devices in an easy and affordable manner [39,44,45].

The aim of this work is to develop 3D printed PCL-based vascular grafts loaded with DIP. The resulting vascular grafts were characterized using different techniques such as such as Fourier-transform infrared (FTIR) spectroscopy, scanning electron microscopy (SEM), differential Scanning Calorimetry (DSC), thermogravimetric analysis (TGA) and X-ray micro-computed tomography. Moreover, the grafts were evaluated by analysing their drug release, antiplatelet effect, hemocompatibility and cytocompatibility.

## 2 Material and methods

### 2.1 Materials

Two different PCL, high molecular weight PCL (CAPA™ 6506, MW = 50,000 g/mol), henceforth referred to as H-PCL, and low molecular weight PCL (CAPA™ 2054, MW = 550 g/mol), henceforth referred to as L-PCL were donated by Perstorp (Malmö, Sweden). DIP was purchased from Tokyo Chemical Industry UK Ltd. (Oxford, UK). Rabbit blood in sodium citrate was provided from Rockland (Reading, UK). Ethanol was provided by Sigma-Aldrich (Dorset, UK). Glutaraldehyde 25% EM Grade was obtained from Agar Scientific Ltd. (Essex, UK). Phosphate buffer solution (PBS) was obtained from VWR Chemicals (Ohio, USA). All materials and reagents were used as received.

### 2.2 Vascular graft design and manufacture

**Fig. 1:**
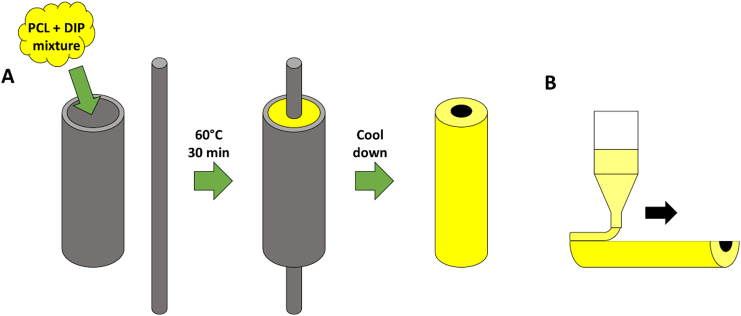
Diagram showing how vascular grafts were prepared using a metal mold (A). Diagram illustrating the 3D printing process (B).

After a variety of preliminary formulation tests (data not shown), a mixture of H-PCL/L-PCL 60%/40% w/w was selected to prepare the different vascular grafts. The PCL mixture was combined with different concentrations of DIP (5%, 10% and 20%) (Table 1) by using the SpeedMixer™ DAC 150.1 FVZ-K (Hauschild GmbH & Co. KG, Westfalen, Germany) at 3000 rpm for 3 min. Then, the mixture was added into a metal mold (Fig. 1) and placed in the oven at 60 °C for 30 min. Immediately after that, a metal bar was used to create the lumen of the grafts (Fig. 1A). Finally, the molded grafts were taken out when the metal mold was cooled down at room temperature.

**Table 1:** Molded grafts formulations.

Formulations	PCL mixture (%) H-PCL (60%)/L-PCL (40%)	DIP (%)
Blank PCL	100	–
5% DIP	95	5
10% DIP	90	10
20% DIP	80	20

#### 2.2.1 3D printing grafts

Two of the previous formulations used for the molded grafts, blank PCL and 20% DIP were also prepared to 3D print vascular grafts. As previously stated, samples were mixed using the SpeedMixer™ DAC 150.1 FVZ-K at 3000 rpm for 3 min prior to the 3D-printing process. Then, the mixture was placed into the metal syringe of the 3D bioprinter at 60 °C for 30 min before printing. The grafts were designed using a computer-aided design (CAD) software and printed using a 3D bioprinter (Bioscaffolder 3.2, GeSiM) (Radeberg, Germany). The 3D BioScaffolder system was equipped with two interchangeable nozzles (0.5 and 0.3 mm). The larger nozzle was used to print objects used during physicho-chemical characterization of the materials. On the other hand, grafts printed for microscopy and computer assisted tomography evaluation were printed using the 0.3 mm nozzle. The print speed was 10 mm/s, the print temperature used was 60 °C and the layer height was 0.35 mm. Finally, these grafts were printed longitudinally as can be seen in Fig. 1B. All layers were aligned in the same direction.

### 2.3 Grafts characterization

The morphology of the designed grafts was evaluated by using scanning electronic microscopy (SEM) (Hitachi TM3030; Tokyo, Japan) and a Leica EZ4 D digital microscope (Leica, Wetzlar, Germany). The digital microscope was equipped with NIGHTSEA Model SFA Stereomicroscope Fluorescence Adapter with the Royal Blue excitation/emission to obtain fluorescence images. To investigate microstructural features of the implants, X-ray microcomputed tomography was performed using a Bruker Skyscan 1275 with Hamamatsu L11871 source at 80 kV and 87 μA. Volumetric reconstruction was performed using Bruker CTvol software, where attenuation thresholding was carried out manually to eliminate speckle around the samples. FTIR spectra of the resulting molded and 3D printing grafts were recorded using a Spectrum Two instrument (Perkin Elmer, Waltham, MA) by the attenuated total reflectance (ATR) technique. The spectra were recorded from 4000 to 600 cm^−1^ with a resolution of 4 cm^−1^ and a total of 32 scans were collected.

The thermal properties of the grafts were evaluated. As the material was subjected to moderate temperatures (60 °C) during the different manufacturing processes, the thermal behavior of the polymer mixtures with and without DIP were examined. For this purpose, a thermogravimetric analysis (TGA) was performed to measure the weight loss of the molded grafts and the 3D printed grafts. Small pieces of these grafts between 3 and 10 mg were used for this analysis. TGA was performed using a Q500 Thermogravimetric analysis (TA instruments, Bellingham, WA, USA). Scans were run from room temperature to 400 °C, at the heating rate of 30 °C/min under a nitrogen flow rate of 50 mL/min. Moreover, A Q100 differential scanning calorimeter (DSC) (TA instruments, Bellingham, USA) was used to establish if the drug was crystalline or amorphous within the resulting grafts. For this purpose, the drug powder and again small pieces of the grafts were examined. Scans were run from 30 °C to 200 °C at 10 °C/min under a nitrogen flow rate of 50 mL/min.

### 2.4 Platelet adhesion study

Blood platelet deposition on the grafts surface was measured using rabbit platelet-rich plasma (PRP), which was produced by centrifuging the rabbit blood in sodium citrate (Rockland Immunochemicals, Inc.; Pottstown, USA) at 1840 rpm for 15 min [46]. Small pieces of 1 mm were obtained from the different molded (Blank PCL and 5, 10 and 20% DIP) and 3D printed grafts (Blank PCL and 20% DIP) and placed in 96-well plate. A 200 μL aliquot of the PRP was poured on the sample surface in the 96-well plate. Samples were then incubated at 37 °C for 2 h. After this incubation step, samples were washed three times using PBS in order to remove the non-adherent platelets. Further, a 2.5% glutaraldehyde solution was used to fix the PRP-treated samples for 2 h. After washing three times with PBS, a series of ethanol solutions (70% for 15 min and 100% for 24 h) were used to dehydrate the samples. Finally, the adhered blood platelets on the grafts surface were observed by using SEM (Hitachi TM3030; Tokyo, Japan).

### 2.5 *In vitro* drug release studies

A release study was performed to calculate the amount of DIP eluting from the resulting molded grafts. For this end, molded grafts of 5 mm in length containing DIP (5%, 10% and 20%) were weighted and placed in vials containing 25 mL of PBS to maintain Sink conditions. Subsequently, these vials were located in a shaking incubator at 37 °C at 40 rpm. At specific time points (each 24 h), the samples were removed from the vials, dried, and transferred to new vials containing 25 mL of fresh PBS. The concentration of DIP was quantified using fluorescence spectroscopy (FLUOstar Omega Microplate Reader, BMG LABTECH, Ortenberg, Germany). For this purpose, the collected release medium was diluted 1:1 in 100% ethanol (as performed in the calibration curve). The fluorescence intensity was then measured at 280 nm (excitation wavelength) and 460 nm (emission wavelength) [1,21].

### 2.6 Mechanical testing

Mechanical testing was carried out to evaluate mechanical properties of 3D-printed blank PCL samples and samples containing 20% of DIP. For this purpose, 1 × 0.7 × 7 cm strips were printed using the same conditions described in Section 2.2.1. and a 0.5 mm nozzle. These strips were printed longitudinally in the same way than the vascular grafts. Mechanical properties were measured using a TA.XTplus texture analyser (Stable Micro Systems, Surrey, UK). Each strip was fixed vertically using two clamps separated 4 cm and stretched at a rate of 10.2 mm/min. Force displacement curves were recorded and different parameters were calculated from these curves. The elastic modulus was obtained as the slope of the initial linear section of the stress/strain curve [47]. Moreover, peak stress and strain were measured as ultimate tensile strength and strain at failure respectively [47].

### 2.7 Hemocompatibility study

Hemolysis was evaluated by determining the relative amount of hemoglobin released from red blood cells (RBCs) in rabbit blood in sodium citrate (Rockland Immunochemicals, Inc.; Pottstown, USA) exposed to the 3D printed constructs [1,48]. Initially, 1 mL of blood was poured into a 1.5 mL Eppendorf tube, which was centrifuged at 2000*g* for 5 min. The supernatant was discarded, and the resulting pellet was resuspended in 1 mL of a saline solution (0.9% NaCl solution). The whole process was repeated 3 times. Finally, the resuspended pellet in 1 mL of saline solution or distilled water (for the positive control) was transferred to a vial containing 9 mL of saline solution or water, respectively. A piece of the 3D printed constructs (weighting between 23 and 32 mg) was placed in a 1.5 mL Eppendorf tube for the hemolytic evaluation. Next, 200 μL of the previous diluted blood solution was added to the tube. After 1 h incubation at 37 °C, the tubes were centrifuged at 2000 *g* for 5 min. Then, the supernatant was carefully pipetted out and transferred into a 96-well plate to measure the absorbance at 545 nm using and UV/vis spectrometer (FLUOstar Omega Microplate Reader, BMG LABTECH, Ortenberg, Germany) [21,49]. The absorbance at 545 nm of the diluted blood with distilled water or saline solution to make positive and negative controls, respectively, was also measured. Finally, the percentage of hemolysis or hemolysis ratio was calculated by Eq. (1).

(1) $$\text{Hemolysis}\;\left(\%\right)=100\times \mathrm{TS}-\mathrm{NC}/\mathrm{PC}-\mathrm{NC}$$

where TS is the absorbance value of the test sample, and NC and PC are the absorbance values of negative and positive controls.

### 2.8 HUVECs growth

3D printed grafts were sterilised under UV irradiation for a total of 1 h and briefly calibrated with warm cell culture medium (EGM-2 media (LONZA 00190860)). To evaluate cellular accommodation and growth, sterilised grafts were placed in a 24 well low-attachment surface culture plate. A total of 500,000 human umbilical vein endothelial cells (HUVECs) (ATCC CRL-1730) were suspended in 500 μL of EGM-2 media and distributed on the top of each graft. The prepared plate was briefly swirled to allow even distribution of the cells in each well before being placed in the incubator. Cells were allowed to attach for a total of 24 h before being supplemented with fresh cell medium. Thereafter cells were cultured for an additional 48 h to assess graft biocompatibility. Cellular attachment was assessed through phase contrast light microscopy, whilst cellular viability and proliferation was assessed through the use of the CyQUANT™ NF Cell Proliferation Assay (Thermo Fisher Scientific, C35006). Before the performance of the cell proliferation assay, each graft was carefully transferred to a new well.

### 2.9 Statistical analysis

All quantitative data were expressed as a mean ± standard deviation. Statistical analysis was performed using a one-way analysis of variance by ANOVA with Tukey's post-hoc. For the cell proliferation experiments and mechanical testing an unpaired two-tailed *t-*test was used. A statistical level of *p* < 0.05 was considered to be statistically significant.

## 3 Results and discussion

### 3.1 Preparation and characterization of PCL-based vascular grafts containing DIP

**Fig. 2:**
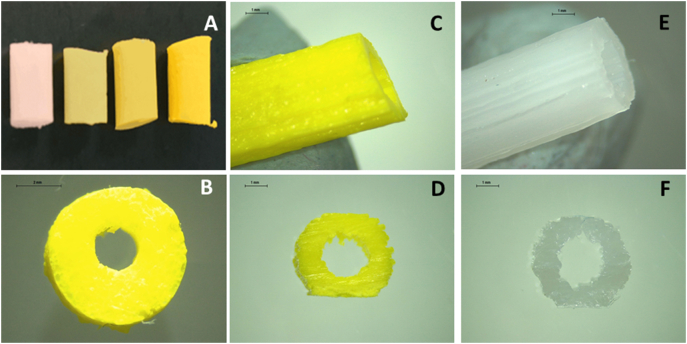
Molded grafts containing no DIP, 5%, 10% and 20% DIP (left to right) (A). Cross section image of the 20% DIP molded graft (B). 3D printed graft containing 20% DIP (C) and its cross section (D). 3D printed graft containing no DIP (E) and its cross section (F). Scale bar panel B: 2 mm. Scale bars panels C–F: 1 mm.

Cylindrical grafts with a diameter of 6 mm containing different DIP concentrations were manufactured using metal mold and 3D printing technology. The molded grafts were prepared to do a preliminary evaluation of the PCL-based materials loaded with DIP. Subsequently, 3D printing was used to prepare vascular grafts using the higher DIP concentration (20% w/w) as preliminary work on DIP loaded materials suggested that higher DIP loadings ranging between 15 and 20% provided the best antiplatelet effect [21]. Additionally, blank grafts were prepared using 3D printing. The yellow color of the grafts darkened as the amount of DIP added increased, confirming that an increased amount of DIP is present in the performed grafts (Fig. 2A). It can be inferred that the drug was mixed successfully with the PCL matrix. This can be corroborated by microscopy (Fig. 2B–F) which shows the drug compound (DIP) was dissolved within the PCL matrix, as the vascular grafts and their cross sections were completely homogeneous, showing no visible DIP aggregates. Thus, it is indicating that the mixing process, a dual asymmetric centrifugal laboratory mixer system, was successfully performed. The good miscibility between PCL and DIP can be explained by their chemical structures. Interactions between the PCL matrix and DIP, possibly through hydrogen bonding due to the hydroxyl groups, which are present in the DIP molecule, may be influencing the mixing process [21,33].

**Fig. 3:**
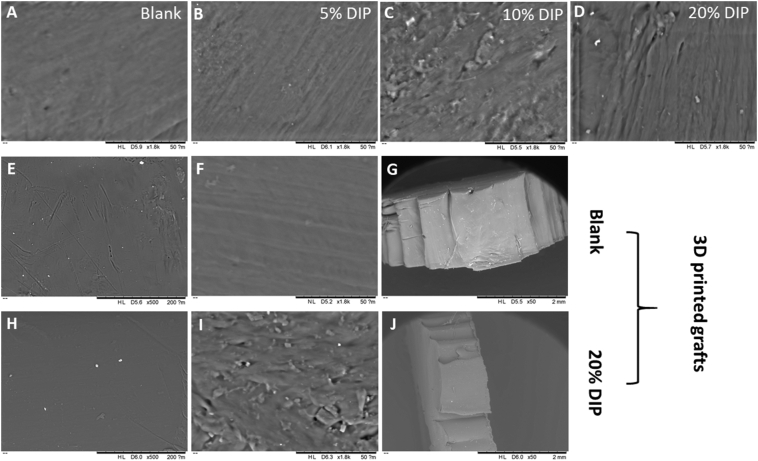
SEM images of the molded grafts containing different DIP concentrations (A–D). SEM images of the 3D printing grafts containing no DIP (E and F) and its cross section (G). Scale bars: 200 μm, 50 μm and 2 mm respectively. SEM images of the 3D printing grafts containing 20% DIP (H and I) and its cross section (J). Scale bars: 200 μm, 50 μm and 2 mm respectively.

The good miscibility between PCL and DIP was also evaluated by using SEM. SEM images of the vascular grafts are shown in Fig. 3 and no visible DIP crystals or aggregates were observed in their surfaces or cross sections. Moreover, the surface and the cross section of the blank samples were quite homogeneous indicating both H-PCL and L-PCL were successfully mixed. Although no visible DIP crystals were found, the surface of the vascular grafts became rough after increasing DIP percentage. As reported in the literature, drug concentration can affect the surface morphology of the final device [21,50].

The use of L-PCL in combination with H-PCL may increase the rate of degradation of the produced implantable devices, as previously reported by Stewart et al. [51]. In fact, degradation time of PCL is dependent on its molecular weight [24]. Higher molecular weight results in a greater number of ester bonds that need to be cleaved [24]. Moreover, L-PCL allowed a better DIP integration within the thermoplastic matrix without using any solvents as required by using electrospinning process. This type of solvents can be harmful to humans, particularly when using for implantable devices such as vascular grafts [1]. In addition, electrospinning technique is more complicated to use and control in comparison with 3D printing techniques. Moreover, one the advantage of this emerging technology is that surgeons can easily design a vascular graft on demand, with modifications in the shape, size and dose, allowing the device to be personalized to the individual needs of each patient [[52], [53], [54], [55]]. These modifications will be more complicated when using electrospinning process.

**Fig. 4:**
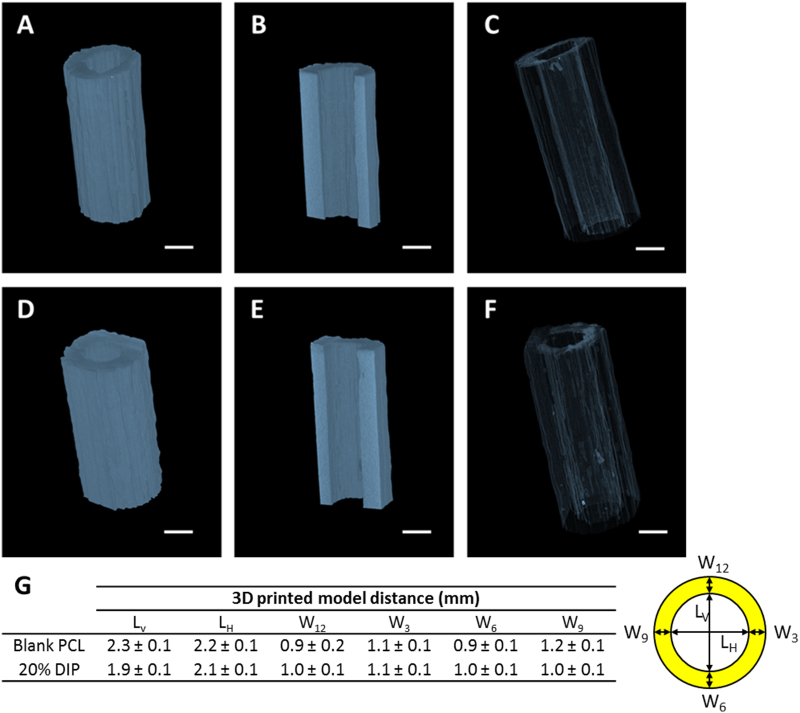
Characterization of the 3D printed blank PCL (A, B, C) and 20% DIP-loaded (D, E, F) vascular grafts through μCT analysis. Representative images of the 3D volume reconstruction (A, D), x-y cross section (B, E) and inner topology (C, F); scale bar = 2 mm. Table showing the dimensions of the 3D printed vascular grafts and the cross-section diagram with legend for these dimensions (G).

The 3D printed vascular grafts were analysed using a Bruker Skyscan 1275 μCT system in order to investigate their architecture and distribution of drug. Both types of samples were characterized by a homogenous and defect-free structure and the presence of DIP did not alter their morphology. As reported in Fig. 4A–F, volumetric reconstructions showed no differences between blank PCL and 20% DIP-loaded vascular grafts. This is most likely due to the similar density values of the polymeric material and drug used, which are both around (1 g/cm^3^). These results indicate that the grafts were well printed and did not present any defects in the graft walls. PCL layers of material were fused together as the printing temperature was higher than PCL melting temperature. Additionally, Fig. 4G shows the dimensions of the 3D printed vascular grafts. These results suggest that the resulting 3D printed grafts presented lumen dimensions *ca.* 2 mm of lumen. These results show that 3D printing can be used for the production of small diameter vascular grafts (<6 mm internal diameter) [13,14]. When comparing the dimensions described in Fig. 4G for blank vascular grafts and 20% DIP vascular grafts it was seen that the only dimension that was significantly different was L_V_. Blank PCL showed a slightly larger values for this dimension. This can be due to the printing process as the grafts were produced by adding layers of materials along that axis. This can be easily corrected when printing by correcting distance between the plate and the nozzle before printing.

**Fig. 5:**
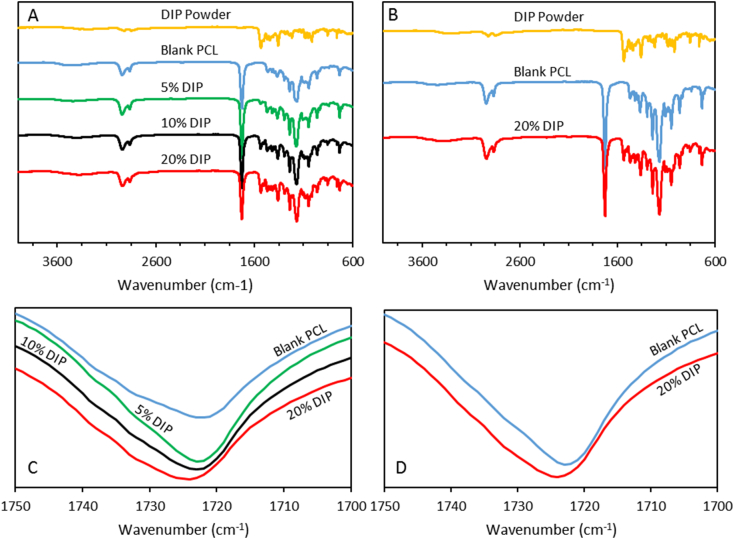
FTIR spectra of the molded (A) and 3D printed grafts (B). Magnification of the carbonyl peak for the molded (C) and 3D printed grafts (D).

The obtained FTIR spectra of the different vascular graft formulations and DIP crystalline powder are presented in the Fig. 5. The spectra of the blank vascular grafts exhibited characteristic peaks at 2943 cm^−1^, 2869 cm^−1^ and 1725 cm^−1^ that can be assigned to the –CH_3_ asymmetric stretching, –CH_3_ symmetric stretching and —C=O stretching, respectively, as has been previously reported [56,57]. Furthermore, the FTIR spectrum of the powder drug revealed characteristic peaks, among others, at 2921 cm^−1^ and at 1530 cm^−1^ that can be attributed to the —CH_3_ asymmetric stretching and —C=N, stretching, respectively [56]. FTIR spectroscopy corroborated that higher amount of DIP is present in the vascular grafts when amount of drug added increased. In the Fig. 5A can be observed that the relative intensity of these DIP characteristic peaks increased in the different DIP-containing formulations when the DIP content was increased. Moreover, these peaks were not seen in the blank formulation. Interestingly, the carbonyl peak of PCL shifts to higher wavenumbers when DIP loading increases (Fig. 5C–D). Moreover, the peak width increases with DIP concentration (Fig. 5C). These results suggest an increase of the amorphous PCL fraction. DIP molecules will be located in between PCL chains reducing the crystallinity of the material. As expected, the same behavior was observed in the 3D printed grafts when DIP was added to the formulation (Fig. 5B).

**Fig. 6:**
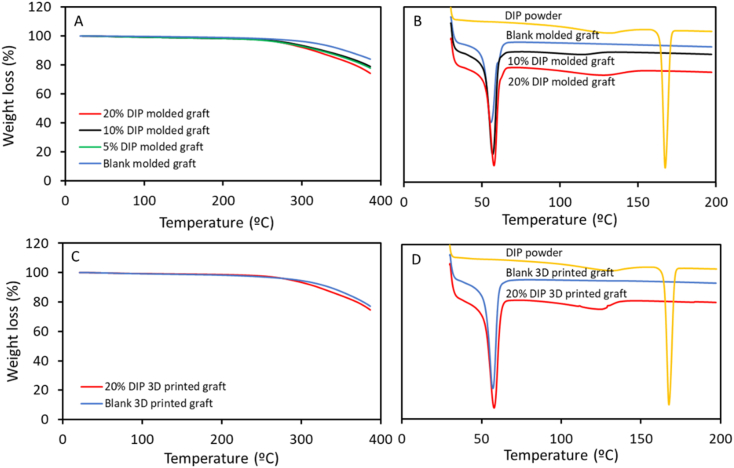
TGA (A) and DSC (B) curves of molded grafts and TGA (C) and DSC (D) curves of the 3D printed grafts.

Thermal analysis was performed to establish if there were any interactions between PCL and DIP, as previously stated. TGA analysis showed that when DIP was incorporated to the PCL-based matrix, the thermal behavior of the resulting materials was different than the one observed for the original PCL-based materials. For both, molded and 3D printed grafts, the *T*_*onset*_ of the PCL was shifted to a lower *T*_*onset*_, when 20% of DIP as added (Fig. 6A and C). To further investigate these interactions between PCL and DIP, DSC analysis was also performed (Fig. 6B and D). The DIP DSC curve showed a sharp endothermic melting point at around 167.5 °C. This peak was not observed in the PCL-based vascular grafts containing DIP suggesting that the drug is interacting within the PCL matrix and thus it is not forming crystals. Similar results have been reported for other types of drugs such as curcumin [58,59]. These results are consistent with the FTIR results suggesting that DIP is interacting with PCL altering the polymer matrix crystallinity. Therefore, these results obtained from thermal analysis are also confirming the previously stated interactions between PCL and DIP.

### 3.2 Platelet adhesion experiment

**Fig. 7:**
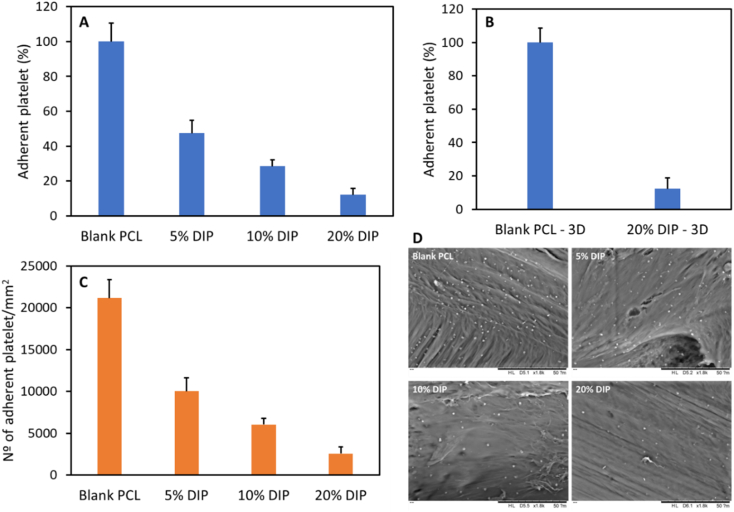
Percentage of rabbit blood platelet depositions on the molded (A) and 3D printed (B) vascular grafts surface; and quantification of rabbit blood platelet depositions on the molded vascular grafts surface (C). SEM images of rabbit blood platelet depositions on the surfaces of the molded vascular grafts (D).

It is well known that when blood comes into contact with a foreign material surface, platelets play a key role in the initiation and propagation of the coagulation cascade, that could lead to the formation of a fibrin clot [19,60]. Therefore, rabbit PRP was used to measure the platelet adhesion on the vascular grafts surface. The results revealed a clear decreased platelet deposition in all the DIP-loaded vascular grafts with respect to the one manufactured from PCL without the drug (Fig. 7). As expected, the molded vascular grafts containing 20% DIP showed the highest reduction in the number of adhered platelets to the material surface (87.89%) followed by the vascular grafts containing 10% and 5% DIP (71.51% and 52.60%, respectively) (Fig. 7A). Significant difference was found between the blank and DIP-loaded vascular grafts. The same results were found in the 3D printed vascular grafts (Fig. 7B). The molded and 3D printed vascular grafts containing 20% DIP showed virtually the same reduction in the number of adhered platelets, 87.89% and 87.67%, respectively, with respect to the PCL grafts (*p* > 0.05). The number of deposited platelet/mm^2^ is also showed in Fig. 7C and D. The reduction in the number of adhered platelets in our DIP-loaded vascular grafts are in line or even better than the ones found using aspirin or DIP-loaded electrospun scaffolds made from PCL or biodegradable elastic poly(urethane), respectively [19,21]. These results confirm that the 3D printed material showed equivalent behavior to the molded grafts containing 20% (w/w) of DIP. Additionally, the results confirm that the selected DIP loading for the 3D printed grafts was appropriate as it provided the best results in terms of platelet adhesion. Accordingly, this drug loading was selected for subsequent experiments. However, before evaluating the biocompatibility of the 3D printed vascular grafts, DIP release over time was measured to evaluate the potential DIP release over the antiplatelet activity.

### 3.3 *In vitro* drug release

**Fig. 8:**
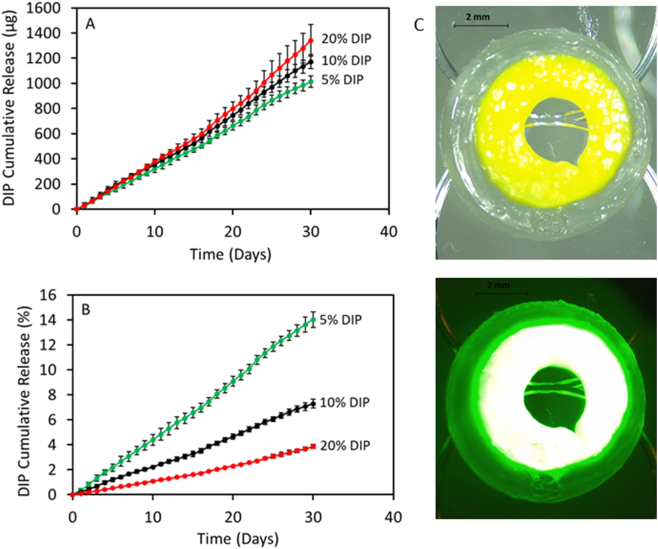
DIP release expressed in μg as a function of time for the different DIP loaded grafts (A). DIP release expressed in percentage as a function of initial DIP drug loading (B) (n = 4). Microscopy image of the cross-section of a core shell implant prepared with 20% DIP in the core and PCL 25 kDa in the shell (C). The graph was printed using 2 extruders from the BioScaffolder equipment: 20% DIP core was printed with a mechanical extruder (nozzle size: 0.50 mm) and the PCL shell printed with pneumatic extruder (nozzle size: 0.25 mm). Bottom panel: fluorescence microscopy image.

In order to ascertain the mechanism of the antiplatelet activity of the materials, DIP release studies were studied for 30 days. This experiment was designed to ascertain if samples containing higher DIP loadings presented a higher drug release to explain the antiplatelet activity. DIP release profiles from DIP-loaded vascular grafts in PBS are shown in Fig. 8. DIP loaded vascular grafts showed a sustained and linear drug release for 30 days without any obvious burst release, or any faster initial release rates for 30 days. Similar results have been reported by Punnakitikashem et al. using DIP loaded nanofibrous scaffolds [21]. However, in this work, the authors obtained release curves showing two clear regions: a first phase with a quicker release during the first 3 days followed by a second slower linear phase. It could be explained by the fact that the solved DIP in the polymer solution had more predisposition to migrate close to the electrospun fibers surface during the electrospinning process, as previously reported for metronidazole benzoate and PCL electrospun nanofibers [61]. Furthermore, the release profile can be controlled by the amount of added DIP to the vascular grafts. As expected, the vascular grafts containing 20% DIP showed the higher drug release (1341.83 μg) followed by the vascular grafts containing 10% and 5% DIP (1172.00 and 1012.86 μg, respectively) (Fig. 8A). On the other hand, when the release is expressed as percentage of the initial DIP loading, the vascular grafts containing 5% DIP showed a higher percentage released (14.03%), followed by the vascular grafts containing 10% and 20% DIP (7.30 and 3.85%, respectively) (Fig. 8B). Therefore, these results also support the previous described interaction between the PCL matrix and DIP. The same behavior has also been reported using a different polymer matrix, thermoplastic polyurethane (TPU), with DIP [1], or other drug compounds, such as levofloxacin and 17-β-estradiol [36,37].

It is important to note that materials containing higher DIP loadings showed a more effective antiplatelet activity. The DIP release results suggest that during the initial stages of the release experiments the resulting materials containing different DIP loadings showed similar release profiles in terms of amount of DIP released. However, the antiplatelet activity reported in Fig. 7 suggest that these 3 types of materials have an antiplatelet activity proportional to DIP loading. These results suggest that the antiplatelet activity of the material is correlated to the DIP loading into the material rather than to the DIP released. Accordingly, these results suggest that in order to achieve good antiplatelet activity the drug should remain in the surface of the material rather than been released. In this way, 20% seem to be the best option not only for the previously reported antiplatelet activity but for its slower release. Fig. 8B shows that materials containing 20% DIP showed slower drug release over a period of 1 month. Accordingly, grafts prepared using this particular drug loading will maintain their antiplatelet activity for prolonged periods.

It is important to note that DIP release will take place over all the surface of the graft. In order to prevent the release of DIP from the outer surface of the material, core-shell grafts can be manufactured. For this purpose a 3D printer equipment equipped with 2 extruders/nozzles can be used [1]. Fig. 8C shows the cross section of this type of implants that have been prepared using a 2-nozzle configuration for this 3D printer. Fluorescence microscopy image shows that DIP is located in the inner part of the graft whilst pure PCL is coating the graft.

### 3.4 Mechanical properties of 3D printed vascular grafts

**Fig. 9:**
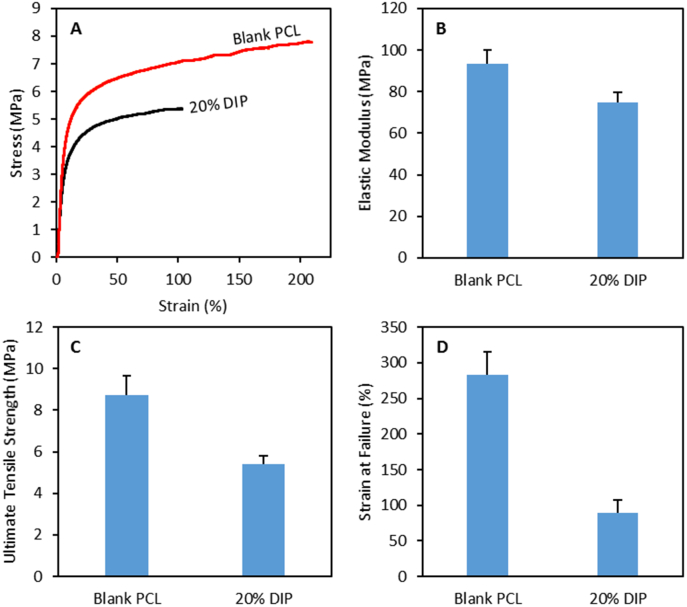
Representative stress/strain curves for 3D printed samples (A). Elastic modulus (B), ultimate tensile strength (C) and strain at failure (D) for 3D printed samples (n = 4).

As discussed in previous sections, grafts loaded with 20% of DIP presented the optimal properties in terms of antiplatelet activity and drug release. Therefore, this type of 3D printed vascular grafts were selected for further investigation. Mechanical properties of the selected 3D printed materials were measured. Fig. 9A shows representative examples of the stress/strain curves obtained for samples containing 0 and 20% (w/w) of DIP. The elastic modulus, ultimate tensile strength and strain at failure were measured (Fig. 9B–D). Blank PCL samples are prepared combining two PCLs with different molecular weights. The elastic modulus of the resulting material is lower than the elastic modulus reported for the high molecular weight PCL alone (93.4 *vs.* 363.4 MPa) [62]. Accordingly, it can be stablished that low molecular weight PCL acts as a plasticiser. Moreover, the results suggest that incorporating DIP into the sample has a direct influence on the mechanical properties of the resulting material. The results obtained for the young modulus indicated that blank samples were stiffer than samples containing 20% DIP (*p* < 0.05). DIP improve the elasticity of the material in the elastic region. This indicates that the drug is interacting with the PCL matrix as suggested in previous sections. However, the presence of DIP affects the plastic behavior of the polymer leading to lower ultimate tensile strengths and strains at failure (*p* < 0.05). Both types of materials presented higher elastic modulus than native blood vessels (0.3–1.5 MPa) [63]. However, these values are lower than the elastic modulus of the materials currently used to prepare synthetic vascular grafts such as: poly(tetrafluoroethylene) (500 MPa) [64] or poly(ethylene terephthalate) (Dacron) (14,000 MPa) [63,64]. Moreover, the elastic modulus of these PCL-based materials are lower than other biodegradable polymers used to prepare vascular grafts: poly(lactic acid) (1000–4000 MPa) or poly(glycolic acid) (7000–10,000 MPa). The elastic modulus of the materials described here are in line with the ones reported for poly(lactic-*co*-glycolic acid) (40–135 MPa) [63]. On the other hand the ultimate tensile strength is not as high as the one obtained for poly(tetrafluoroethylene) (14 MPa) or poly(ethylene terephthalate) (170–180 MPa) [63]. However, the obtained values are in line with the majority of native blood vessels (1.4–11.1 MPa) or other materials such as poly(lactic-co-glycolic acid) [63]. Finally, the ultimate tensile strength of the materials is superior to the one reported for native blood vessels (17–105%) [64].

### 3.5 Hemolytic evaluation

**Fig. 10:**
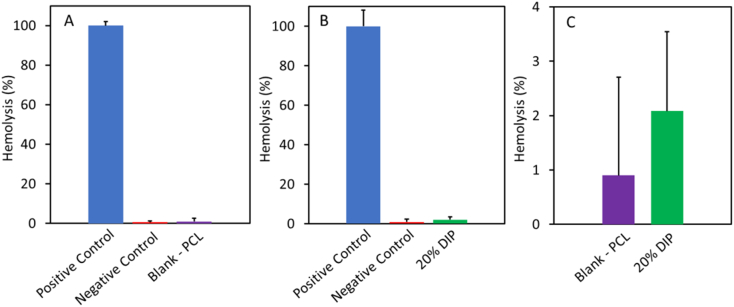
Rabbit blood hemolysis percentages of the 3D printed vascular grafts, blank PCL (A) and 20% DIP (B). Magnification of the results obtained from the 3D printed vascular grafts (C) (n = 4).

The next step after characterizing the grafts was to evaluate the hemocompatibility of the resulting grafts. This test is important, as vascular grafts are medical devices that are going to be in direct contact with blood. For this test both 3D printed grafts (Blank PCL and 20% DIP) were evaluated. The hemolysis percentage of both were lower than 4% (Fig. 10). Although the vascular grafts containing 20% DIP showed a higher hemolysis percentage, there was no significance difference with respect to the blank PCL (*p* > 0.05). Samples displaying a hemolysis percentage lower than 5% have been defined as hemocompatible [65,66].

### 3.6 *In vitro* HUVECs growth

**Fig. 11:**
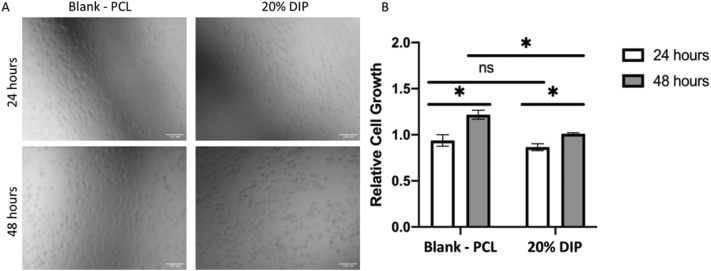
HUVEC attachment on the surfaces of Blank-PCL and DIP 20% loaded materials 24 and 48 h post seeding. Scale bar = 100 μm (A). HUVEC proliferation inhibition was assessed using a CyQUANT™ NF Cell Proliferation Assay after incubation for up to a total of 48 h, following a prior 24 h period of time allotted to allow for cellular attachment post seeding. The PCL graft without loaded drugs 24 h were set as the control. Error bars represent mean ± SEM (n = 3), *p* value shown: *p* < 0.05. (B).

The biocompatibility of each 3D printed vascular graft (Blank PCL and 20% DIP) was investigated through culturing HUVECs onto UV-sterilised grafts for a total of 72 h and assessing cell viability and proliferation. Following an incubation period of 24 h, cells were found to readily attach to each graft. Evaluation 24 h post the incubation period through phase contrast microscopy, as can be seen in Fig. 11A, confirmed the HUVECs to have formed a monolayer on each graft type. Further analysis 48 h post incubation displayed each graft type to host an even greater number of cells compared to 24 h, as was supported by the CyQUANT NF cell proliferation assay (Fig. 11B) (*p* > 0.05). However, whilst the cell proliferation assay did not detect a significant difference between the two grafts at the 24-h time point, a statistically significant increase in growth was observed between the two grafts at the 48-h timepoint, suggesting the cells to proliferate more readily on the control graft. Nevertheless, each graft was able to provide a suitable milieu to stimulate a significant increase in cell growth between the two time points. Overall, this data collectively indicates the ability of the 20% DIP loaded grafts to provide a supportive environment for cellular attachment, viability, and growth.

## 4 Conclusions

Biodegradable PCL-based vascular grafts loaded with DIP were successfully manufactured using 3D printing technology. DIP was selected as a drug candidate due mainly to its antithrombotic effect. DIP was properly mixed within the PCL matrix, which was confirmed by SEM. This good miscibility can be attributed to the hydrogen bonding between the drug and the polymer matrix. Moreover, the results of the thermal analysis are also corroborating an interaction between DIP and the PCL matrix. DIP-loaded grafts are capable of providing a sustained and linear drug release without any obvious burst release, or any faster initial release rates for 30 days. These results could be explained by the previously stated hydrogen bonding and hydrophobic interactions between DIP and PCL. The resulting DIP-loaded grafts showed a clear antithrombotic effect compared with the ones made from PCL without the drug. The reduction in the number of deposited platelets to the material surface increased by increasing the amount of DIP loaded in the grafts. In addition, all the materials prepared in this work were hemocompatible and cytocompatible. The obtained results suggest that 3D printing can be successfully used for this purpose, and therefore has a great potential to be transferred to clinical applications. Moreover, this emerging technology allows the manufacture of medical devices on demand, with modifications in the shape, size and dose, allowing the devices to be personalized/designed to the individual needs of each patient. However, there are several aspects that will need to be evaluated prior to use this technology for clinical applications including *in vivo* animal experiments. Moreover, key aspects such as the sterilisation of this type of devices need to be evaluated. Conventional techniques can be used for this purpose such as gamma irradiation and ethylene oxide. However, this will need to be addressed carefully as some sterilisation techniques can alter the properties of PCL-based vascular grafts [67]. Finally, it is important to note than before any 3D printing technology can be used for medical device manufacturing regulatory clearance should be obtained.

## CRediT authorship contribution statement

**Juan Domínguez-Robles:** Methodology, Investigation, Formal analysis, Writing – original draft, Writing – review & editing. **Tingjun Shen:** Methodology, Investigation, Formal analysis, Writing – original draft. **Victoria A. Cornelius:** Investigation, Formal analysis, Writing – original draft. **Francesca Corduas:** Investigation. **Elena Mancuso:** Supervision, Investigation, Writing – original draft, Writing – review & editing. **Ryan F. Donnelly:** Funding acquisition. **Andriana Margariti:** Supervision. **Dimitrios A. Lamprou:** Writing – review & editing. **Eneko Larrañeta:** Supervision, Conceptualization, Methodology, Funding acquisition, Writing – review & editing.

## Declaration of competing interest

The authors declare that they have no known competing financial interests or personal relationships that could have appeared to influence the work reported in this paper.
